# Analysis of risk factors and construction of a predictive model for contralateral occult carcinoma in patients with unilateral papillary thyroid carcinoma

**DOI:** 10.3389/fendo.2025.1532840

**Published:** 2025-04-24

**Authors:** ShiYi Zhao, Wei Yan, Jin Yu, DeJie Chen

**Affiliations:** ^1^ College of Medicine, Wuhan University of Science and Technology, Wuhan, China; ^2^ Department of Thyroid and Breast Surgery, Xiangyang Central Hospital, Affiliated Hospital of Hubei University of Arts and Science, Xiangyang, China

**Keywords:** papillary thyroid cancer (PTC), occult cancer, predictive modelling, total thyroidectomy (TT), risk factor

## Abstract

**Objective:**

This study aims to investigate the risk factors associated with contralateral occult carcinoma in patients with unilateral papillary thyroid carcinoma and to develop a corresponding prediction model to enhance early detection and clinical management of occult carcinoma.

**Methods:**

The clinical data of 430 patients who underwent total thyroidectomy for unilateral papillary thyroid carcinoma at Xiangyang Central Hospital between January 2021 and December 2022 were collected. Univariate and multivariate logistic regression analyses were performed to identify risk factors for contralateral occult cancer in patients with unilateral thyroid carcinoma. A prediction model was established, and the diagnostic value of the model was assessed using calibration curves and decision curve analysis.

**Results:**

The results of univariate logistic regression analysis indicated that tumor diameter, tumor location, multifocality, presence of contralateral benign nodules, and lateral neck lymph node metastasis were risk factors for contralateral occult carcinoma in patients with unilateral thyroid cancer (P < 0.05). Multivariate logistic regression analysis further showed that a tumor diameter >1 cm, proximity of the tumor to the isthmus, multifocality, presence of contralateral benign nodules, and lateral neck lymph node metastasis were independent risk factors for contralateral occult carcinoma in unilateral thyroid cancer (P < 0.01). A risk nomogram model was developed based on these five risk factors, with areas under the curve (AUC) of 0.921 and 0.96 for the training and validation sets, respectively. The calibration curve demonstrated good consistency, and decision curve analysis indicated that the model had a high level of net benefit.

**Conclusion:**

A tumor diameter >1 cm, proximity of the tumor to the isthmus, lateral neck lymph node metastasis, presence of contralateral benign nodules, and multifocality are independent risk factors for contralateral occult carcinoma in patients with unilateral papillary thyroid carcinoma. The predictive model developed in this study demonstrates strong predictive ability for the occurrence of contralateral occult carcinoma in patients with unilateral papillary thyroid carcinoma.

## 1 Introduction

Thyroid cancer is the most common endocrine malignancy, with papillary thyroid carcinoma (papillary thyroid cancer, PTC) being the predominant subtype (1). In recent years, the incidence of thyroid cancer has been steadily increasing, with an annual growth rate as high as 20.1% in China (2), Despite its relatively low aggressiveness and generally favorable prognosis (3, 4), the presence of contralateral occult carcinoma in certain patients can significantly impact treatment and long-term management (5–8). Understanding the characteristics of occult carcinoma and the challenges in its diagnosis is therefore of paramount importance.

Occult carcinoma refers to a tumor focus that remains undetected through preoperative examinations such as ultrasonography or fine-needle aspiration, only to be confirmed postoperatively through pathological evaluation. Previous studies have suggested that the occurrence of occult carcinoma may be closely associated with various clinical factors (2, 9–11). Identifying these high-risk factors is crucial for developing personalized treatment plans, optimizing surgical strategies, and improving patient outcomes.

Although previous studies have attempted to construct predictive models for contralateral occult carcinoma, these models still lack accuracy and clinical applicability. Traditional methods, such as multivariate regression analysis and simple scoring systems, can identify some risk factors but fail to comprehensively validate the diagnostic value of the models, particularly without incorporating modern analytical tools like calibration curves and decision curve analysis (7, 9). This study addresses these limitations by introducing nomograms and decision curve analysis, significantly enhancing the predictive accuracy of the model (AUC = 0.96) and validating its potential clinical utility.

Against this backdrop, this study, based on a large and up-to-date dataset, focuses on identifying high-risk factors for contralateral occult carcinoma in patients with unilateral papillary thyroid carcinoma. It also constructs a predictive model aimed at enabling early intervention and helping clinicians more accurately identify high-risk patients. This model allows physicians to formulate more precise preoperative treatment plans, thereby optimizing the management and long-term outcomes of thyroid cancer.

## 2 Materials and methods

### 2.1 General information

The clinical data of 430 patients who underwent total thyroidectomy for the treatment of papillary thyroid carcinoma (PTC) at Xiangyang Central Hospital between January 2021 and December 2022 were retrospectively collected. The inclusion criteria were as follows:(1). First-time surgery with the surgical procedure being total or near-total thyroidectomy; (2).Postoperative pathological diagnosis confirming bilateral PTC; (3).Preoperative ultrasound or fine-needle aspiration identifying malignancy only on one side; (4).Preoperative ultrasound examination of the thyroid and cervical lymph nodes with complete records and images; (5).Complete documentation of personal information, surgical records, and other clinical data.

Patients were excluded from the study if they met any of the following conditions: (1).Presence of another type of malignancy (other tumors) before thyroidectomy; (2).Diffuse thyroid disease; (3).Distant metastasis confirmed through pathological or clinical analysis; (4).Pediatric or adolescent patients with a history of neck radiation or a family history of cancer; (5).Special populations, including children and pregnant women; (6).Incomplete clinical data or missed follow-up.

### 2.2 Data collection

The collected data included patient age, gender, presence of preoperative Hashimoto’s thyroiditis, intraoperative tumor location, and postoperative pathological findings such as tumor diameter, multifocality of the primary tumor, thyroidal extracapsular invasion (involvement of surrounding soft tissue, muscles, or blood vessels), lymphovascular invasion, thyroid capsule invasion, central lymph node metastasis, lateral cervical lymph node metastasis, and the presence of benign nodules in the contralateral lobe. The patients included in the study were randomly divided into a training group and a test group in a 7:3 ratio.

### 2.3 Statistical methods

Categorical variables were expressed as frequencies (percentages), and comparisons between groups were performed using the χ² test. Continuous variables were tested for normality; those conforming to a normal distribution were expressed as mean ± standard deviation (x̄ ± s) and analyzed using the t-test, while non-normally distributed data were expressed as median (interquartile range) [M(Q1, Q3)] and analyzed using the Mann-Whitney U test. Risk factors were analyzed using univariate logistic regression, and multivariate logistic regression was performed to control for confounding factors.

The dataset was divided into a training set and a test set to optimize the sensitivity and specificity of the model. A nomogram was constructed to visually display high-risk factors, and the accuracy and clinical utility of the model were evaluated using calibration plots, ROC curves (AUC), and decision curve analysis. These methods provided robust data support for identifying high-risk factors and constructing the predictive model.

## 3 Result

As shown in **Table 1**, the study included a total of 430 patients’ basic clinical information, with the incidence of contralateral occult PTC being 18.6% (80 cases). The collected data included gender, tumor size, location of the primary tumor, presence of thyroid extracapsular invasion, lymphovascular invasion, capsular invasion, central lymph node metastasis, benign nodules in the contralateral lobe, lateral cervical lymph node metastasis, presence of Hashimoto’s thyroiditis, and contralateral occult carcinoma. In this retrospective study, all variables had p-values greater than 0.05, indicating no significant differences between the training and test sets in these features. The baseline characteristics were similar, demonstrating high comparability between the two groups. This suggests that differences between groups primarily stemmed from interventions or other variables rather than differences in baseline characteristics.

**Table 1:** Comparison of demographic and clinical pathological characteristics of 430 patients.

Variable	Group		P
	Test Set (n = 129)	Training Set (n = 301)	
Contralateral Occult Carcinoma, n (%)			0.499
Yes	27 (21.9)	53 (17.6)	
No	102 (79.1)	248 (82.4)	
Age, n (%)			0.603
<55 years	91 (70.5)	203 (67.4)	
≥55 years	38 (29.5)	98 (32.6)	
Gender, n (%)			0.499
Male	21 (16.3)	59 (19.6)	
Female	108 (83.7)	242 (80.4)	
Tumor Diameter, n (%)			0.883
≤1 cm	66 (51.2)	158 (52.5)	
>1 cm	63 (48.8)	143 (47.5)	
Tumor Location, n (%)			1
Isthmus	24 (18.6)	55 (18.3)	
Isthmus and Others	105 (81.4)	246 (81.7)	
Multifocality, n (%)			0.508
Yes	57 (44.2)	121 (40.2)	
No	72 (55.8)	180 (59.8)	
Contralateral Benign Nodules, n (%)			0.144
Yes	65 (50.4)	127 (42.2)	
No	64 (49.6)	174 (57.8)	
Central Lymph Node Metastasis, n (%)			0.566
Yes	77 (59.7)	169 (56.1)	
No	52 (40.3)	132 (43.9)	
Lateral Cervical Lymph Node Metastasis, n (%)			0.873
Yes	26 (20.2)	57 (18.9)	
No	103 (79.8)	244 (81.1)	
Extracapsular Invasion, n (%)			0.978
Yes	24 (18.6)	54 (17.9)	
No	105 (81.4)	247 (82.1)	
Lymphovascular Invasion, n (%)			1
Yes	0 (0.0)	1 (0.3)	
No	129 (100.0)	300 (99.7)	
Capsular Invasion, n (%)			0.077
Yes	71 (55.0)	136 (45.2)	
No	58 (45.0)	165 (54.8)	
Hashimoto’s Thyroiditis, n (%)			0.661
Present	40(31.0)	87(28.9)	
Absent	89(69.0)	214(71.1)	

As presented in **Table 2**, univariate logistic regression analysis revealed that “tumor diameter” (*P* = 0.019), “tumor location” (*P* <0.001), “multifocality” (*P*<0.001), “contralateral benign nodules” (*P*<0.001) and “lateral cervical lymph node metastasis” (*P*<0.001) were significantly associated with prognosis (*P* < 0.05). In contrast, variables such as extracapsular invasion (*P* = 0.172), lymphovascular invasion (*P* = 1), capsular invasion (*P* = 0.232), central lymph node metastasis (*P* = 0.324), and Hashimoto’s thyroiditis (*P* = 0.82) showed no significant association with prognosis (*P* > 0.05). These findings provide a basis for multivariate analysis and suggest the potential predictive value of the significant factors identified for risk stratification.

**Table 2:** Univariate logistic analysis of the training set.

	B	Standard Error	Wald	Degrees of Freedom	Significance	Exp(B)	95% Confidence Interval of Exp(B)	
							Lower Limit	Upper Limit
Age	0.278	0.315	0.782	1	0.376	1.321	0.713	2.447
Gender	-0.087	0.375	0.054	1	0.816	0.916	0.439	1.911
Tumor Diameter	0.728	0.311	5.477	1	0.019	2.07	1.126	3.807
Tumor Location	1.531	0.336	20.809	1	< 0.001	4.624	2.395	8.927
Multifocality	1.617	0.333	23.526	1	< 0.001	5.036	2.621	9.678
Contralateral Benign Nodules	0.994	0.312	10.149	1	0.001	2.703	1.466	4.984
Central Lymph Node Metastasis	0.307	0.311	0.973	1	0.324	1.359	0.739	2.499
Lateral Cervical Lymph Node Metastasis	1.685	0.333	25.551	1	< 0.001	5.392	2.806	10.362
Extracapsular Invasion	-0.631	0.462	1.864	1	0.172	0.532	0.215	1.316
Lymphovascular Invasion	-19.664	40192.97	0	1	1	0	0	0
Capsular Invasion	-0.372	0.311	1.431	1	0.232	0.69	0.375	1.268
Hashimoto’s Thyroiditis	0.075	0.331	0.052	1	0.82	1.078	0.564	2.061

As shown in **Table 3**, the clinical and pathological indicators with significant differences in the univariate logistic regression analysis were included in the multivariate logistic regression analysis. The results indicated that several variables were significantly associated with risk. Specifically, tumor diameter >1 cm (OR = 9.562, *P* < 0.001), isthmic lesions (OR = 13.467, *P* < 0.001), multifocal lesions (OR = 35.847, *P* < 0.001), contralateral benign nodules (OR = 15.582, *P* < 0.001), and lateral cervical lymph node metastasis (OR = 23.829, *P* < 0.001) were identified as independent risk factors.

**Table 3:** Multivariate logistic analysis of the training set.

	B	Standard Error	Wald	Degrees of Freedom	Significance	Exp(B)	95% Confidence Interval of Exp(B)	
							Lower Limit	Upper Limit
Tumor Diameter > 1 cm	2.258	0.509	19.686	1	< 0.001	9.562	3.527	25.925
Isthmus	2.6	0.529	24.171	1	< 0.001	13.467	4.776	37.971
Multifocality	3.579	0.616	33.733	1	< 0.001	35.847	10.712	119.956
Contralateral Benign Nodules	2.746	0.508	29.17	1	< 0.001	15.582	5.752	42.209
Lateral Cervical Lymph Node Metastasis	3.171	0.548	33.51	1	< 0.001	23.829	8.144	69.719

**Figure 1:**
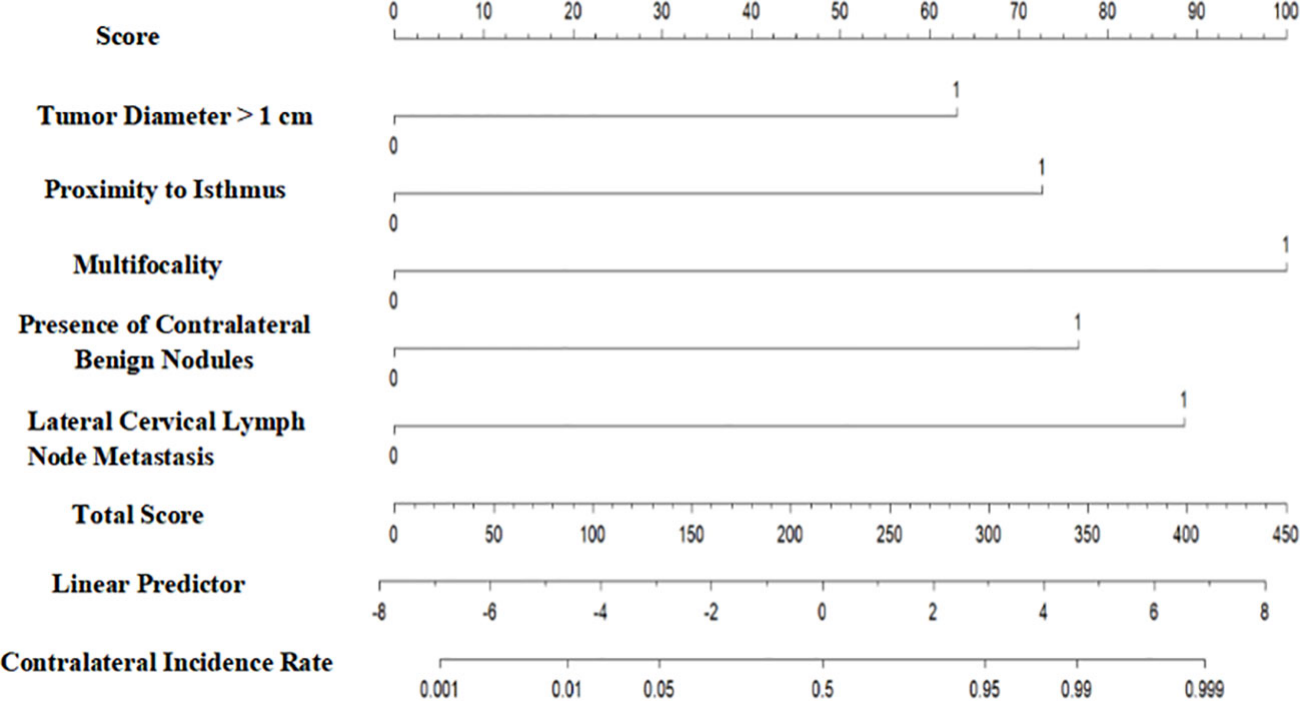
Incidence rate of contralateral occult carcinoma in the thyroid.

As illustrated in **Figure 1**, the cancer-specific survival prediction nomogram integrates multiple prognostic variables, including tumor diameter >1 cm, proximity of the tumor to the isthmus, multifocality, presence of contralateral benign nodules, and lateral cervical lymph node metastasis. Each variable is assigned a specific score to quantify its impact on patient prognosis. The sum of the variable scores constitutes a total score, with higher total scores indicating poorer prognosis. The total score is further converted into the probability of contralateral occult carcinoma occurrence in thyroid cancer using a linear predictor.

**Figure 2:**
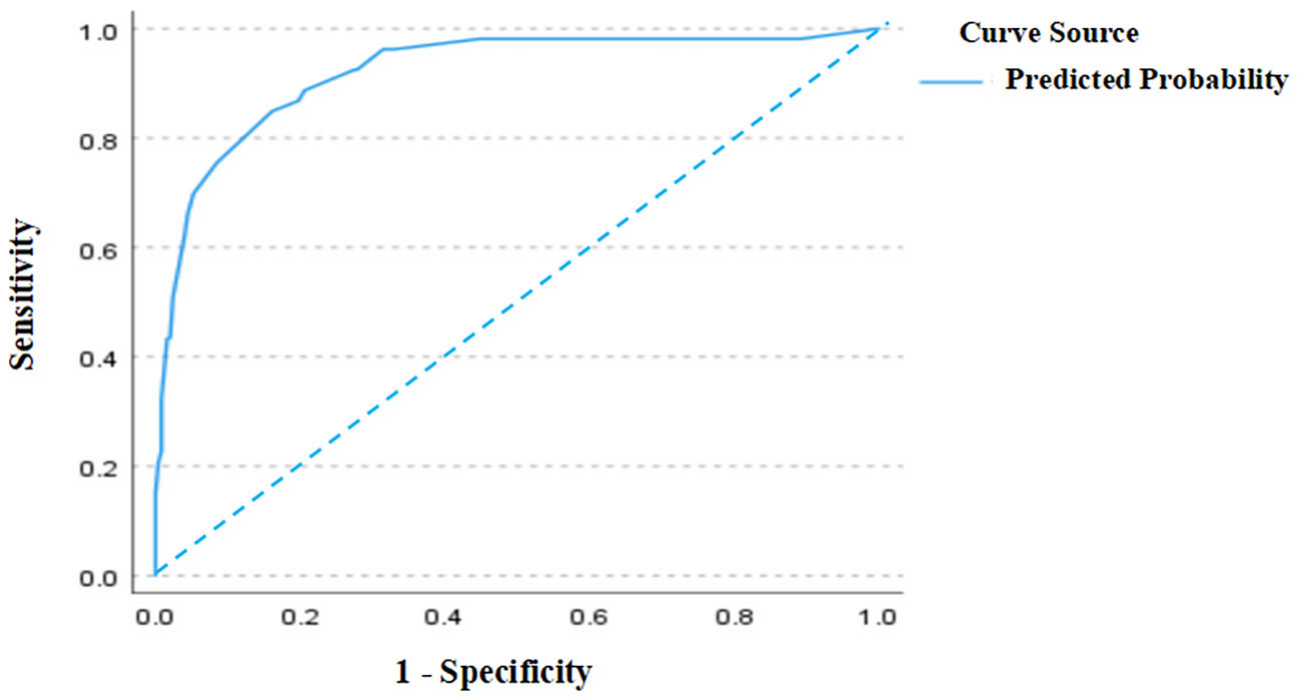
ROC curve (training set).

**Figure 3:**
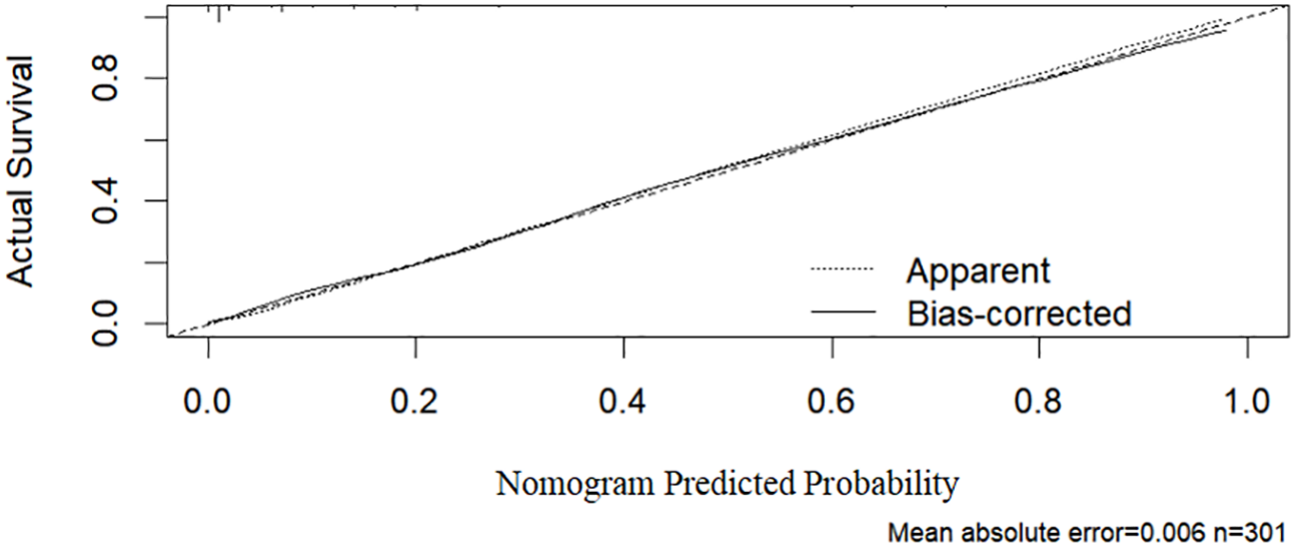
Calibration curve (training set).

As shown in **Figure 2**, the ROC curve analysis of the training set revealed an AUC of 0.921 (95% CI: 0.877–0.964), indicating excellent discriminative ability. The standard error was 0.022, and *P* < 0.001, demonstrating that the model significantly outperforms random classification. In **Figure 3**, the calibration curve shows a high degree of agreement between predicted probabilities and actual probabilities, with the bias-corrected curve closely aligning with the diagonal. The mean absolute error was 0.006, indicating good calibration performance in the training set, with high precision and stability.

**Figure 4:**
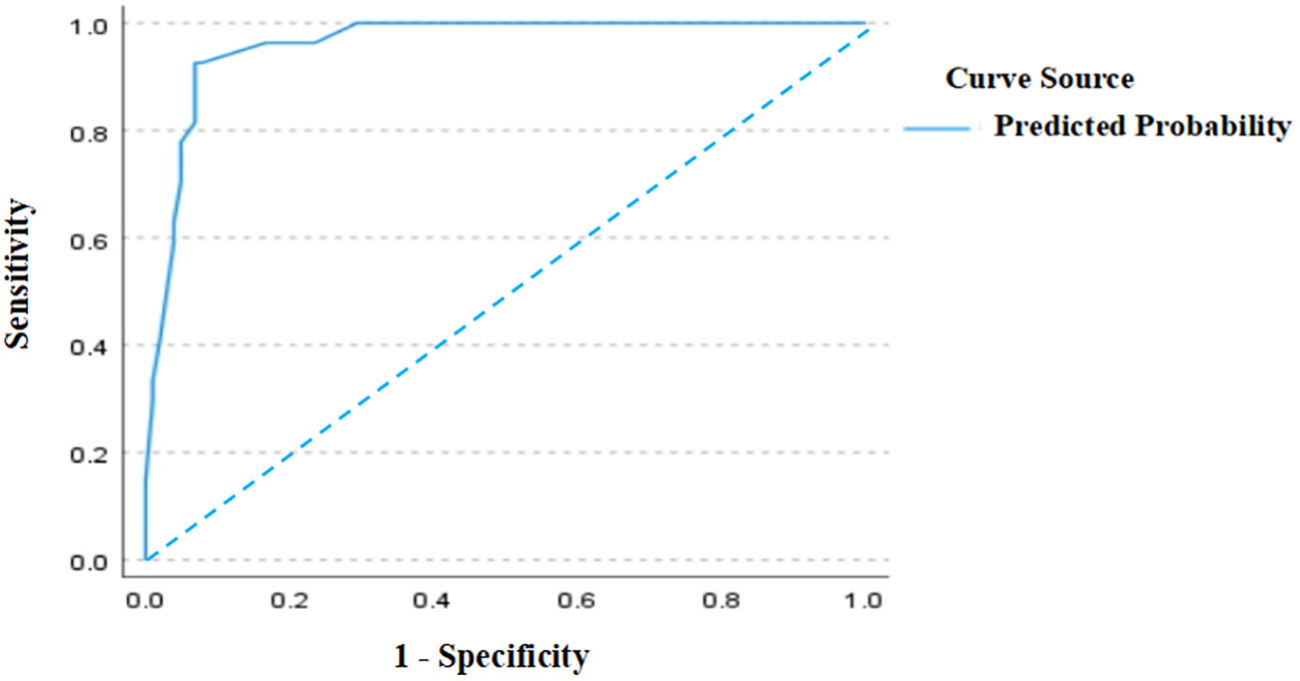
ROC curve (test set).

**Figure 5:**
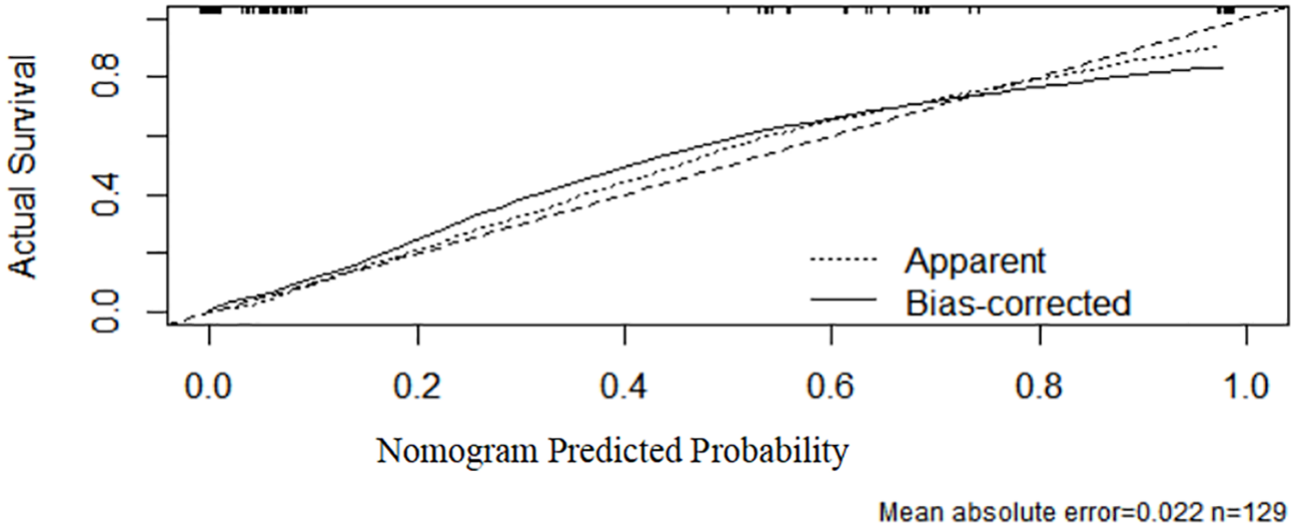
Calibration curve (test set).

As shown in **Figure 4**, the ROC curve analysis of the test set demonstrated an AUC of 0.96 (95% CI: 0.928–0.992), verifying the model’s superior predictive performance on unseen data and exhibiting strong generalization capability. The standard error was 0.016, and *P* < 0.001, confirming significant discriminative efficacy. These results further support the model’s efficiency and reliability in the test set. In **Figure 5**, the calibration curve of the test set shows that the bias-corrected curve closely aligns with the diagonal, indicating good agreement between predicted and actual probabilities. However, compared to the training set, the calibration in the test set showed a slight decline, with a mean absolute error of 0.022, indicating minor stability reduction. The sample size of the test set was 129.

**Figure 6:**
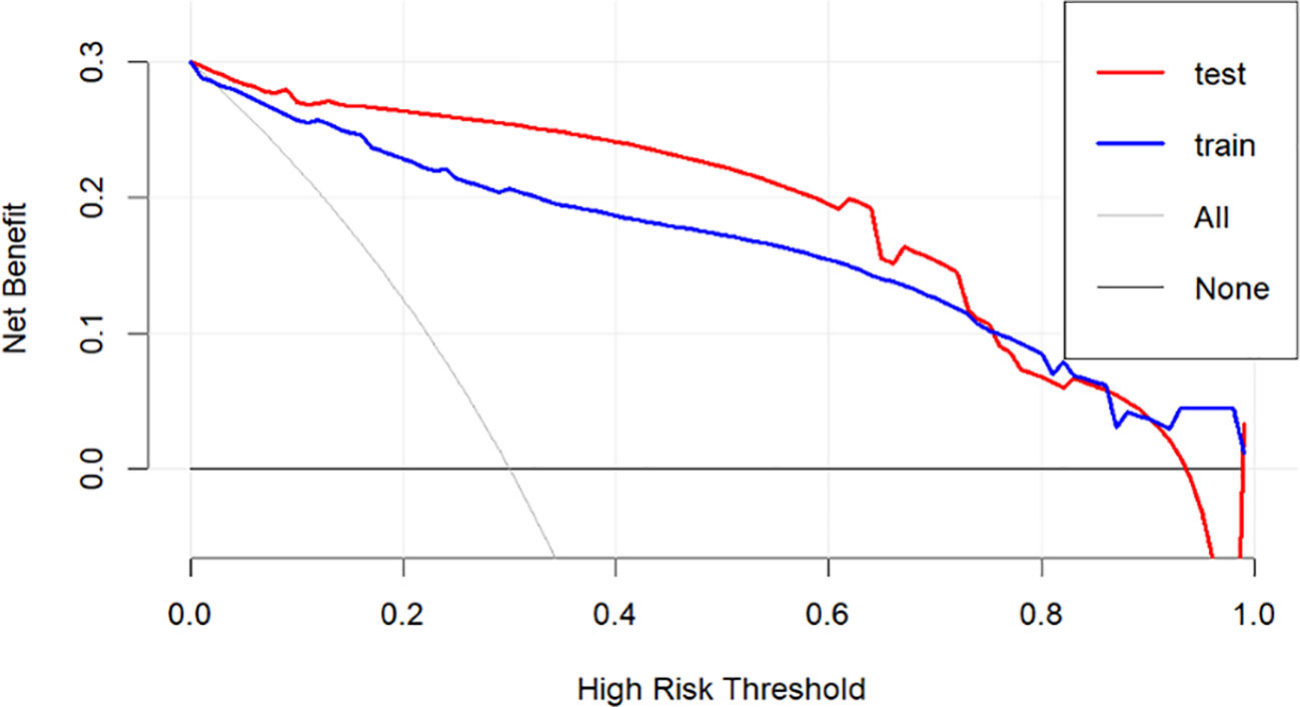
Decision curve analysis (DCA).

In **Figure 6**, the decision curve analysis (DCA) evaluates the net benefit of the model across different risk thresholds. The x-axis represents high-risk thresholds, while the y-axis represents net benefit. The figure includes four curves: the red curve represents the test set net benefit, the blue curve represents the training set net benefit, the gray curve (All) assumes all patients are high-risk, and the black curve (None) assumes all patients are low-risk. The analysis shows that the net benefit curves for both the test and training sets are higher than the gray and black lines across most threshold ranges, indicating that the model outperforms the assumptions of all-high-risk or all-low-risk scenarios and has clinical decision-making value. Particularly in the low-to-moderate risk range, the model achieves higher net benefit, which gradually declines as the threshold increases, approaching the black line. This suggests reduced clinical value of the model at higher thresholds.

## 4 Discussion

Papillary thyroid carcinoma (PTC) is a common thyroid malignancy with a significantly rising incidence in recent years, attracting extensive attention and in-depth research (2). With advancements in medical technology, the understanding of the pathological characteristics and prognostic factors of thyroid cancer has been continuously refined. This study identifies a series of factors closely associated with the occurrence of contralateral occult carcinoma, including tumor diameter >1 cm, lateral cervical lymph node metastasis, tumor location near the isthmus, multifocality, and the presence of contralateral benign nodules. These findings provide critical guidance for clinical practice.

Multiple studies have shown that a tumor diameter >1 cm is a significant predictor of contralateral occult carcinoma (10–13). Larger tumors are generally proportional to their aggressiveness, which is not only reflected in their growth rate but also in their metastatic potential (10). Larger tumors are often associated with a higher likelihood of lymph node metastasis and more extensive tissue invasion, laying the foundation for the development of contralateral occult carcinoma and highlighting the critical role of tumor size in treatment decision-making.

In a retrospective study involving 573 patients, Feng et al. (9) pointed out that tumor location is not associated with the occurrence of contralateral occult cancer. However, in this study, tumor proximity to the isthmus was identified as a high-risk factor for the occurrence of contralateral occult carcinoma. Tumors in the isthmus, being located near both thyroid lobes, may directly affect thyroid structure and function, increasing the risk of contralateral carcinoma. Additionally, the lymphatic drainage pathways of the isthmus may differ from those of the upper pole, potentially contributing to the risk of occult carcinoma, which has not been adequately studied. Although existing literature has paid limited attention to this issue, it provides a direction for future research and underscores the need for an in-depth exploration of the relationship between isthmic tumors and contralateral occult carcinoma.

Additionally, lateral cervical lymph node metastasis and multifocality significantly increase the risk of occult carcinoma, consistent with previous studies (7, 13–17). Multifocal tumors indicate that the malignancy may grow in multiple foci within the thyroid. A meta-analysis reported that multifocal tumors have nearly double the risk of lymph node metastasis compared to solitary tumors (18). Consequently, clinical guidelines generally recommend total or near-total thyroidectomy for patients with multifocal primary PTC, regardless of staging.

This study also demonstrates that the presence of contralateral benign nodules may be a risk factor for contralateral occult carcinoma, consistent with prior findings (19). Benign nodules may obscure contralateral occult malignancies, making it challenging to detect potential malignant lesions through imaging studies. Therefore, when evaluating thyroid nodules, clinicians should pay particular attention to contralateral benign nodules and consider more detailed examinations to ensure occult carcinoma is not overlooked.

Although several high-risk factors for contralateral occult carcinoma were identified, this study found that gender, age, central lymph node metastasis, capsular invasion, perithyroidal invasion, lymphovascular invasion, and Hashimoto’s thyroiditis were not significantly associated with its occurrence. These findings are clinically important as they simplify the risk assessment process, allowing clinicians to focus on factors that genuinely influence the risk of occult carcinoma.

Gender and age, while common influencing factors in cancer incidence and prognosis (20, 21), were not found to have a direct association with the occurrence of contralateral occult carcinoma in this study. This may be attributed to the complexity of patient characteristics and limitations in sample size. Additionally, pathological features such as capsular invasion, perithyroidal invasion, and lymphovascular invasion, which are closely related to prognosis in many cancer types (22–24) showed minimal impact on occult carcinoma, likely due to the relatively indolent biological behavior and lower aggressiveness of occult carcinoma compared to other thyroid cancer subtypes.

Based on the identified high-risk factors, this study developed a predictive model for contralateral occult carcinoma. The model enables clinicians to devise more proactive treatment strategies for high-risk patients, such as total thyroidectomy and lymph node dissection. Furthermore, the model can play a critical role in postoperative follow-up by helping determine which patients require more frequent monitoring and examinations to detect potential recurrence or metastasis promptly.

This study has several limitations. First, as a single-center retrospective study, it is inherently subject to selection bias, which may limit the external validity and generalizability of the findings. Although the sample size was relatively large, certain potential variables, including molecular biomarkers such as BRAF V600E, NRAS, and e mutations, as well as environmental factors like iodine intake, were not included in the analysis. The omission of these factors may result in the exclusion of significant risk determinants. The lack of molecular data was primarily due to the financial constraints of patients, the high cost of genetic testing, and its exclusion from routine medical insurance coverage, making it impractical to include as part of standard clinical evaluations. Additionally, limited patient awareness of the clinical significance and utility of genetic testing further reduced acceptance rates, which constrained the availability of relevant data. Given these circumstances, this study focused on clinical and pathological characteristics to identify the risk factors for contralateral occult carcinoma in patients with unilateral papillary thyroid carcinoma. This approach reflects the pragmatic consideration of resource limitations and patient circumstances, ensuring the study’s clinical relevance and applicability. However, future studies should prioritize multicenter prospective designs that incorporate molecular genetic data (e.g., BRAF V600E, NRAS) alongside environmental exposures, to generate more comprehensive datasets and validate the generalizability and predictive utility of the identified high-risk factors and predictive model.

In conclusion, this study highlights the importance of identifying high-risk factors for contralateral occult carcinoma and establishing effective predictive models for its clinical management. These findings provide scientific support for preoperative decision-making and patient management while laying the foundation for improving patient prognosis and quality of life. By integrating new research findings and clinical experience, we can better address the growing public health challenge posed by thyroid cancer and provide more effective treatment strategies for patients.

Ethical Statement: This study is a retrospective clinical study conducted in accordance with the principles of the Declaration of Helsinki. All patient data were anonymized and de-identified to protect privacy and confidentiality. As there was no direct patient involvement, the requirement for informed consent was waived by the Medical Ethics Committee of Xiangyang Central Hospital.

## Data Availability

The raw data supporting the conclusions of this article will be made available by the authors, without undue reservation.
